# mTOR Kinase: A Possible Pharmacological Target in the Management of Chronic Pain

**DOI:** 10.1155/2015/394257

**Published:** 2015-01-01

**Authors:** Lucia Lisi, Paola Aceto, Pierluigi Navarra, Cinzia Dello Russo

**Affiliations:** ^1^Institute of Pharmacology, Catholic University Medical School, Largo F. Vito 1, 00168 Rome, Italy; ^2^Department of Anesthesiology and Intensive Care, A. Gemelli Hospital, Catholic University Medical School, 00168 Rome, Italy

## Abstract

Chronic pain represents a major public health problem worldwide. Current pharmacological treatments for chronic pain syndromes, including neuropathic pain, are only partially effective, with significant pain relief achieved in 40–60% of patients. Recent studies suggest that the mammalian target of rapamycin (mTOR) kinase and downstream effectors may be implicated in the development of chronic inflammatory, neuropathic, and cancer pain. The expression and activity of mTOR have been detected in peripheral and central regions involved in pain transmission. mTOR immunoreactivity was found in primary sensory axons, in dorsal root ganglia (DRG), and in dorsal horn neurons. This kinase is a master regulator of protein synthesis, and it is critically involved in the regulation of several neuronal functions, including the synaptic plasticity that is a major mechanism leading to the development of chronic pain. Enhanced activation of this pathway is present in different experimental models of chronic pain. Consistently, pharmacological inhibition of the kinase activity turned out to have significant antinociceptive effects in several experimental models of inflammatory and neuropathic pain. We will review the main evidence from animal and human studies supporting the hypothesis that mTOR may be a novel pharmacological target for the management of chronic pain.

## 1. Introduction

Chronic pain represents a major public health problem worldwide, affecting approximately 37% of the US population, with an economic burden of up to US$ 635 billion per year [1]. In Europe, the prevalence of chronic pain syndromes ranges between 25 and 30% [2]. Physiologically, nociceptive pathways are activated in response to traumatic or noxious stimuli. Acute pain, which is primarily due to nociception, serves as an adaptive and protective mechanism to detect, localize, and limit tissue damage; on the contrary, chronic pain, which persists after a reasonable time for healing to occur (ranging between 1 and 6 months in most definitions), can be regarded as a form of maladaptive response, in which pain is no longer protective or strictly linked to the initial stimulus. After application of an intense and prolonged injury, ongoing excitation of primary nociceptive neurons leads to neuronal changes both in the primary afferents (peripheral sensitization) and in the spinal dorsal horn neurons (central sensitization), contributing to the development of chronic pain [3]. In this condition, pain arises in the absence of noxious stimulus, may be stimulated by normally innocuous stimuli (allodynia), is exaggerated and prolonged in response to noxious stimuli (primary hyperalgesia), and spreads beyond the site of injury (secondary hyperalgesia) [3]. Chronic pain has a neuropathic origin in approximately 20% of the patients [2]. Neuropathic pain may arise from a direct damage of somatosensory nerves or nerves innervating visceral organs or from a disease affecting the somatosensory nervous system which implies an indirect injury resulting from various causes, including metabolic stress, autoimmune, degenerative, or chronic inflammatory conditions, and idiopathic origins [4].

Neuropathic pain is characterized by pain hypersensitivity that is mediated by both peripheral and spinal neuronal synaptic plasticity (leading toperipheral and central sensitization, resp.), involving pre- and posttranslational changes in the expression and functions of receptors, enzymes, and voltage-dependent ion channels in sensory neurons [3]. In addition, other biochemical events contribute to the hyperactivity of the somatosensory system, including phenotypic neuronal switch (i.e., large myelinated A*β* fibers expressing neuropeptides directly involved in pain transmission, such as substance P and calcitonin gene-related peptide), sprouting of nerve endings (i.e., myelinated A*β* fibers establishing direct contacts with nociceptive projecting neurons in the lamina I-II of the spinal dorsal horn), loss of spinal inhibitory control, and increased activity of descending excitatory pathways [3]. Moreover, synaptic plasticity within key cortical regions involved in pain processing (i.e., the anterior cingulated cortex, the insular cortex, primary and secondary sensory cortices, and the amygdala) has been also observed in relation to neuropathic pain [4]. Finally, activation of glial cells with release of pronociceptive mediators can directly modulate neuronal excitability and thus pain transmission, contributing to central sensitization and to the occurrence of neuropathic pain [5].

Multimodal pharmacological treatments for chronic pain syndromes, including neuropathic pain, are based on the use of antiepileptics, antidepressants, local anesthetics, opioid analgesics, or tramadol. These treatments are only partially effective, with significant pain relief achieved in 40–60% of patients [4]. A relatively recent modality of neuropathic pain therapy, which represents the future challenge of upcoming researches, involves specific cellular targets implied in neuronal synaptic plasticity and/or glial activation [6]. Interestingly, recent studies show that the mammalian target of rapamycin (mTOR) kinase and downstream effectors may be implicated in the development of chronic inflammatory, neuropathic, and cancer pain. This kinase is a master regulator of protein synthesis, and it is critically involved in the regulation of several neuronal functions, including synaptic plasticity and memory formation in the central nervous system (CNS) [7]. As mentioned above, neuronal synaptic plasticity both at peripheral level and in the CNS is a major mechanism leading to the development of chronic pain, thus suggesting that mTOR may be a novel pharmacological target for the management of chronic pain. In addition, mTOR has been also reported to regulate astrocyte and microglial activity (as we have recently reviewed [8]), thus suggesting an additional therapeutic target in the treatment of chronic pain syndromes that involve increased glial activation. The main evidence from animal studies as well as clinical reports supporting this hypothesis is reviewed in the present paper.

## 2. mTOR, the “Mechanistic” Target of Rapamycin

**Figure 1:**
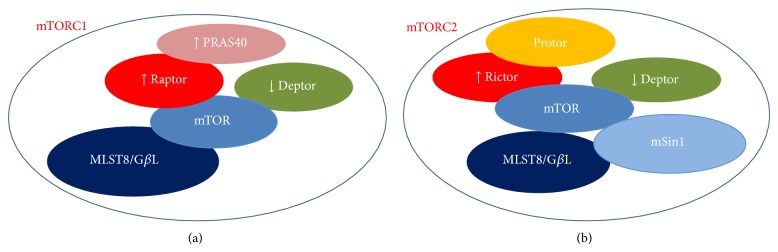
Schematic representing the molecular partners of mTOR forming (a) mTOR complex 1 (mTORC1) and (b) mTOR complex 2 (mTORC2). The down-arrows indicate the inhibitory proteins, whereas the up-arrows indicate activator factors on mTOR function.

The mTOR kinase, now officially known as “mechanistic” TOR, is a conserved serine/threonine protein kinase belonging to the phosphoinositide 3-kinase (PI3K) family that regulates multiple intracellular processes [9]. In mammals, mTOR is encoded by a single gene [10] and interacts with several proteins to form two distinct complexes, referred to as mTORC1 and mTORC2. These complexes display different sensitivity to the inhibitory action of rapamycin, which mainly suppresses mTORC1-dependent activities in acute treatments [11]. Notably, the two complexes promote the activation of different signalling pathways and recognize distinct upstream regulators as well as downstream targets, whose specificity is determined by the specificity of the interacting proteins. Both complexes include the inhibitory protein DEPTOR and the adaptor protein mLST8/G*β*L (mammalian LST8/G-protein *β*-subunit like protein) [12]. However, the role of mLST8/G*β*L in the regulation of mTORC1 function remains unclear at present, since its chronic loss does not affect mTORC1 activity* in vivo* [13]. As shown in Figure 1, mTORC1 specifically contains the regulatory-associated protein of mTOR (raptor) and the inhibitory protein PRAS40 (proline-rich AKT substrate of 40 KDa) [14]. Raptor regulates mTORC1 assembly and serves as a scaffold for the recruitment of specific substrates, such as the eukaryotic initiation factor-4E-binding protein 1 (4E-BP1) [15, 16]. Similarly, other proteins reside uniquely within complex 2, that is, the rapamycin-insensitive companion of mTOR (rictor), the protein observed with rictor (PROTOR), and the stress-activated protein kinase-interacting protein 1 (mSIN1) (Figure 1) [17]. Much like raptor, rictor is necessary for mTORC2 assembly together with mSIN1, for mTORC2 catalytic activity, and it may also be involved in the selective recruitment of specific substrates [14].

**Figure 2:**
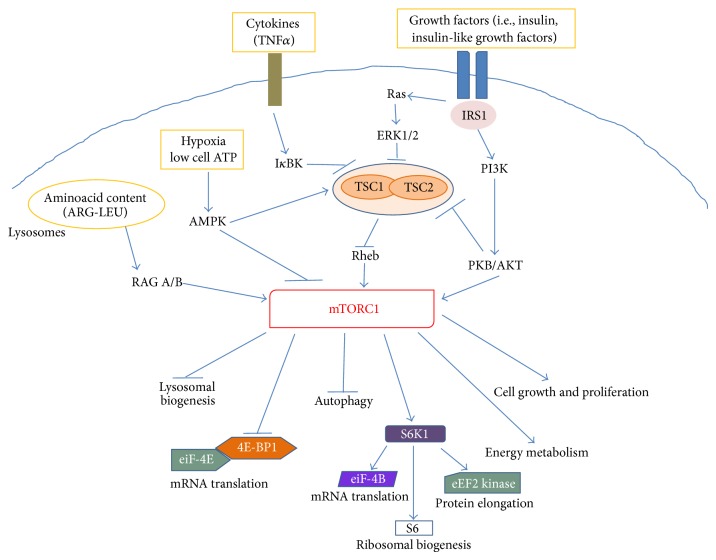
Schematic representing the main intracellular targets as well as the main cellular processes regulated by mTORC1.

The mTOR complex 1 is activated in response to different intracellular and extracellular cues, that is, growth factors, cytokines, energy status, oxygen, and amino acids, to control multiple functions related to cell growth and metabolism [12]. As shown in Figure 2, several upstream regulators of mTORC1 activity converge on theheterodimer consisting of tuberous sclerosis 1 (TSC1, also known as hamartin) and TSC2 (also known as tuberin). In this complex, TSC1 stabilizes TSC2 by preventing its degradation, while TSC2 acts as a GTPase-activating protein (GAP) for the small GTPase protein, Rheb (Ras homolog enriched in brain) [18]. Rheb in its GTP-bound state binds to and activates mTORC1, whereas the TSC1/2 complex normally inhibits mTORC1 activity by favoring the GDP-bound inactive state of Rheb. As recently reviewed by Laplante and Sabatini [12], growth factors (such as insulin and insulin-like growth factors) increase mTORC1 activity, by promoting the phosphorylation and degradation of the TSC1/2 complex. This occurs via ligand-dependent activation of receptor tyrosine kinases (RTKs), like the insulin receptors, followed by activation of the PI3K and Ras pathways. The effector kinases of these pathways, namely, the protein kinase B (PKB/AKT) and the extracellular-signal-regulated kinases (ERK) 1 and 2, induce TSC1/2 phosphorylation (Figure 2). In addition, AKT can directly phosphorylate the inhibitory protein PRAS40, promoting its dissociation from raptor and further contributing to mTORC1 activation. Proinflammatory cytokines, like TNF*α*, increase mTORC1 activity via I*κ*B kinase- (IKK-) dependent inactivation of the TSC1/2 complex, whereas, in response to hypoxia or low energy status, the adenosine monophosphate activated kinase (AMPK) blocks mTORC1 activity, by increasing TSC2 function and directly inhibiting raptor (Figure 2). Finally, increased intracellular levels of aminoacids, particularly arginine and leucine, promote mTORC1 activation, by inducing its binding to a distinct family of GTPases, the Rag GTPases, together with its translocation to the lysosomal surface [19, 20]. It has been hypothesized that translocation of mTORC1 to the lysosomes allows GTP-bound Rheb to interact with mTORC1, promoting its activation only when aminoacids are available. Additional details on the regulation of mTORC1 can be retrieved in the above mentioned review article [12].

Protein synthesis is the best-characterized intracellular process regulated by mTORC1, whose activation generally increases the cellular capacity of protein generation [14]. The two main downstream targets of mTORC1, 4E-BP1 and the ACG-family protein, S6 kinase 1 (S6K1), are key components of the protein translation machinery. Phosphorylation of 4E-BP1 causes its dissociation from the eukaryotic translation initiation factor- (eIF-) 4E (Figure 2). This allows eIF-4E to associate with eIF-4G leading to the formation of eIF-4F, which facilitates the loading of ribosomes onto the mRNA. By this molecular mechanism, mTORC1 can control the translation of specific mRNAs, including the so-called 5′-TOP mRNAs that mostly encode for components of the translational machinery. In addition, phosphorylation and activation of S6K1 promote protein translation by phosphorylation of several substrates, including eIF-4B, the eukaryotic elongation factor 2 (eEF2) kinase, and the ribosomal S6 protein (Figure 2). Local protein synthesis within sensory neurons contributes to their nociceptive functions both under physiological conditions and during chronic pain. As described in detail in Section 4, by controlling protein translation mTORC1 can regulate the activity of sensory neurons, in periphery as well as in the CNS. The activity of S6K1 is also important in the control of RTK activation. In fact, S6K1 (activated by mTORC1) promotes also the phosphorylation and inactivation of IRS1, the insulin receptor substrate 1. The latter is a docking protein that in its tyrosine-phosphorylated form couples the insulin receptor to its downstream effectors [21]. This is part of a retroinhibitory feedback mechanism that reduce RTK activation, thus the activity of AKT and ERK [12]. The latter is also a kinase critically involved in the regulation of pain processing (see Section 4). The mTOR complex 1 is also involved in the regulation of several metabolic pathways, regulating the expression of genes encoding different steps of glycolysis and the pentose phosphate pathway, as well as critical enzymes in the* de novo* biosynthesis of lipids [22]. Finally, mTORC1 can favor cell growth by negatively regulating macroautophagy (autophagy), the central degradative process in cells, and lysosome biogenesis [12, 14]. As discussed in detail in Section 4, the activation of autophagy in Schwann cells can limit the extent of axonal degeneration after nerve injury and promote regeneration and myelination, thus favoring analgesic effects.

**Figure 3:**
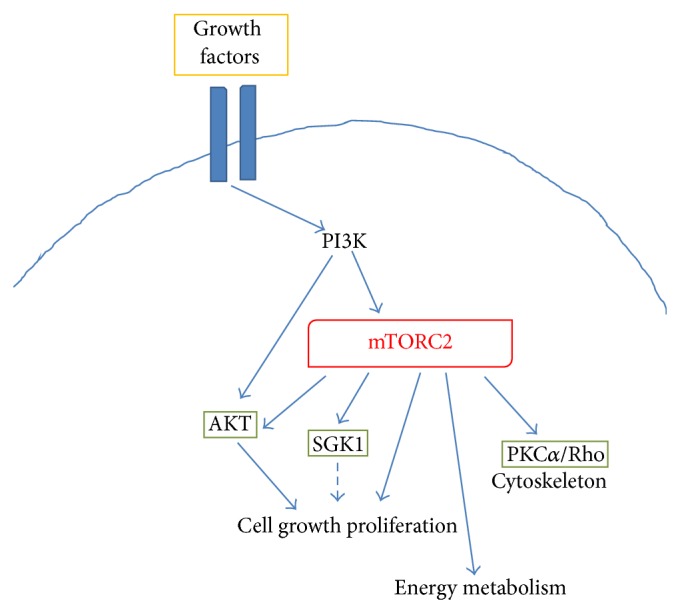
Schematic representing the main intracellular targets as well as the main cellular processes regulated by mTORC2.

**Figure 4:**
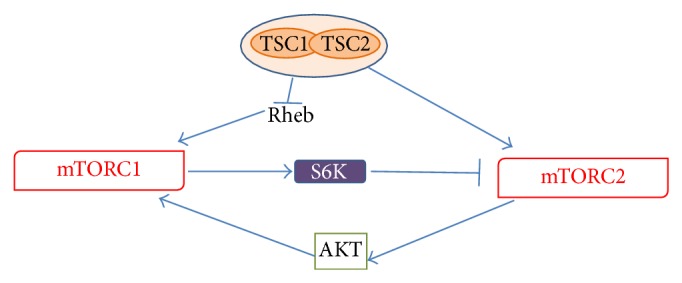
Schematic representing the main mTORC1-mTORC2 crosstalks.

In contrast to mTORC1, mechanisms leading to mTORC2 activation are less characterized. It seems that mTORC2 activation is directly promoted by PI3K via phosphorylation of specific mTORC2 interactors, including rictor (Figure 3) [17]. Thus, mTORC2 appears to be also responsive to growth factors, but insensitive to nutrients. mTORC2 regulates the activity of several proteins belonging to the ACG family, including AKT, the serum- and glucocorticoid-induced protein kinase (SGK1), and protein kinase C- (PKC-) *α*. AKT is a key regulator of cell survival and proliferation, with mTORC2 promoting its phosphorylation at Ser_473_ in the hydrophobic motif and maximal activation [23, 24]. In this way, mTOR appears to be both a downstream effector of AKT (i.e., mTORC1) and an important upstream regulator of the kinase activity (i.e., mTORC2) (Figure 4). Moreover, it has been shown that an intricate crosstalk exists between the two complexes (Figure 4), since S6K can negatively control the activation of mTORC2 [24], whereas TSC1/2 positively contributes to mTORC2 activation [25]. The mTOR complex 2 also promotes the activity of SGK1, another important kinase in the control of cell proliferation [26]. Finally, activation of PKC*α* by mTORC2, along with other effectors (like paxillin and Rho GTPases), regulates the dynamic of actin cytoskeleton [27, 28]. Direct regulation of AKT activity together with a role in the control of actin dynamics suggests a possible involvement of mTORC2 in the control of neuronal function, as discussed in Section 4.

## 3. mTOR Inhibitors

The mTOR kinase, now officially known as “mechanistic” TOR, was initially identified as “mammalian target of rapamycin,” because the kinase is the main target of an antifungal compound derived from* Streptomyces hygroscopicus*, rapamycin [29]. This drug, discovered in soil samples collected from Easter Island (Rapa Nui, from where the name), was originally found to have antifungal proprieties, but rapidly its immunosuppressive activity became its more important property. Actually, rapamycin is widely used in preventing clinical allograft rejection and in treating some autoimmune diseases [30]. In the 1980s, rapamycin was also found to have anticancer activity, although the exact mechanism of action remained unknown until many years later.

Rapamycin (or sirolimus) mainly inhibits mTORC1 activity by forming a trimolecular complex with mTOR and the immunophilin, FKBP12 (FK506-binding protein of 12 kDa; also known as PPIase FKBP1A). The drug associates with FKBP12, and the resulting complex interacts with the FRB (FKBP12-rapamycin binding) domain located in the carboxyl terminus of mTOR: the interaction disrupts the association with raptor and thus uncouples mTORC1 from its substrates inducing a block of mTORC1 signaling [31, 32]. However, not all the functions mediated by mTORC1 are sensitive to rapamycin; the inhibition of cap-dependent translation and the induction of autophagy are in part resistant to rapamycin [33]. Originally, the effects of rapamycin were thought to be only related to the inhibition of mTORC1, but studies of Sabatini's group have shown that rapamycin given at higher concentrations and in chronic treatments also interferes with mTORC2 regulatory functions [11]. In particular, high intracellular levels of rapamycin inhibit the binding and subsequent assembly of mTORC2-specific components mSIN1 and rictor [11].

Rapamycin readily crosses the blood brain barrier (BBB), thus exerting direct effects within the CNS [34]. However, in order to ameliorate the pharmacokinetic profile of rapamycin, novel drugs have been developed. This first generation of mTOR inhibitors displays the same binding sites for mTOR and FKBP12 and is thus so-called rapalogs (i.e., rapamycin and its analogs). Rapalogs includes CCI-779 (temsirolimus), RAD-001 (everolimus), and AP23573 (ridaforolimus or deforolimus) (Table 1). Among these mTOR inhibitors, CCI-779 is a prodrug of rapamycin, which delays tumor proliferation [35], and it is actually used for the treatment of renal cell carcinoma, whereas RAD-001, a 40-O-(2-hydroxyethyl) derivative of rapamycin, is currently used as an immunosuppressant to prevent rejection of organ transplants and, like CCI-779, for the treatment of renal cell cancer and subependymal giant cell astrocytoma [36].

However, the use of rapalogs unmasked the feedback loop between mTORC1 and AKT in certain type of cells. The mTORC1 inhibition induced by these drugs fails to repress the negative feedback loop that results in phosphorylation and activation of AKT, and it is unable to block the mTORC2 positive feedback to AKT [37]. The elevation of AKT activity can promote a longer survival in some cell types and may also be associated to pain hypersensitivity (as described in Section 4). These limitations have led to the development of a second generation of mTOR inhibitors: the ATP-competitive mTOR inhibitors, which block both mTORC1 and mTORC2 activity [38]. Unlike rapamycin, which is a specific allosteric inhibitor of mTORC1, these ATP-competitive inhibitors target the catalytic site of the enzyme, thus promoting a broader, more potent, and sustained inhibition of mTOR and preventing the activation of PI3K/AKT caused by the derepression of negative feedbacks [39]. This is due to the effective inhibition of rapamycin-insensitive mTORC2 activity in addition to mTORC1 inhibition and also to a more comprehensive and sustained mTORC1 inhibition as demonstrated by sustained reduction of 4E-BP1 phosphorylation [38]. Actually, many compounds with different chemical structures show these functions (see Table 1) and some of them are being tested in clinical trials.

Finally, the close interaction of mTOR with the PI3K pathway has also led to the development of mTOR/PI3K dual inhibitors [40]. Compared with drugs that inhibit either mTORC1 or PI3K, these drugs have the benefit of inhibiting both mTORC1 and mTORC2 and all the catalytic isoforms of PI3K. Interestingly, because of the high sequence homology between mTOR and PI3K, some compounds (like wortmannin), originally identified as PI3K inhibitors, were later shown to inhibit mTOR as well [41]. The activity of these small molecules differs from rapalog activity, for a more specific block of both mTORC1-dependent phosphorylation of S6K1 and mTORC2-dependent phosphorylation of AKT at the Ser_473_ residue. Dual mTOR/PI3K inhibitors include NVP-BEZ235, BGT226, SF1126, and PKI-587 (Table 1), and many of them are being tested in early-stage of preclinical trials.

A detailed list of mTOR inhibitor drugs is provided in Table 1, together with specific information on their molecular properties (including* in vitro* mTOR IC50 and cellular potency towards mTORC1 and mTORC2). Dual mTORC1/mTORC2 inhibitors have been developed by counterscreening their inhibitory activity against the most closely related kinases, class I and class III PI3K lipid kinases and the PI3K-related kinase (PIKK) family members [42], whereas dual PI3K/mTORC inhibitors were optimized to inhibit class I PI3Ks [43]. Thus, the* in vitro* inhibitory potency against class I PI3Ks has been also included in Table 1. As far as the ATP-competitive mTOR inhibitors (first generation and second generation) are concerned, we calculated the ratio between PI3K and mTOR IC50 and marked those drugs with a ratio <500 since inhibition of PI3K by these drugs may become relevant in cellular or* in vivo* systems. This information should provide the reader with a better understanding of the biological effects of these novel drugs.

## 4. mTOR in the Control of Chronic Pain

### 4.1. Histology

The expression and activity of mTOR have been extensively detected in peripheral and in central regions involved in pain transmission, both under physiological conditions and in several experimental models of inflammatory and neuropathic pain. In the adult rat and mouse cutaneous tissue, mTOR immunoreactivity was found in a subset of primary sensory axons and in nonneuronal cells surrounding the peripheral axons in the dermis [44, 45]. Using specific markers that distinguish between C- and A-fibers, it has been shown that mTOR positive axons are mainly myelinated A-fibers, and only less than 5% are peptidergic fibers, coexpressing CGRP. Interestingly, these fibers were stained also for the active form of mTOR, evaluated by measurement of mTOR phosphorylation at Ser_2448_. This phosphorylation primarily reflects a feedback signal from the mTORC1 downstream target S6K1 (also known as p70S6 kinase, p70S6K), and it is therefore considered a reliable marker of mTORC1 activation within the cells [46]. Moreover, myelinated sensory fibers in the rat skin also express phosphorylated downstream targets of mTORC1, including 4E-BP1, S6K, and the S6 ribosomal protein [44], suggesting that mTORC1 may regulate local protein synthesis within these axons thus contributing to their nociceptive functions under physiological conditions. These fibers, particularly the large A-beta (A*β*) fibers, are normally involved in the conduction of nonnociceptive inputs such as light touch, movement, or vibration [47]. However, amplification of their signals by sensitized dorsal horn neurons is thought to account for secondary hyperalgesia and allodynia, clinical features of chronic pain (as summarized in Section 1). Consistently, active mTORC1 and its downstream phosphorylated targets (4E-BP1 and the S6 ribosomal protein) have been also detected in the adult rat dorsal roots, mostly in myelinated axons [48]. Local or intrathecal administration of rapamycin significantly reduced phosphorylation of downstream targets of mTORC1 both in the peripheral fibers [44] and in the central spinal cord neurons [48]. Similarly to these data, phospho-mTOR was also found to be expressed by a subset of myelinated fibers in the adult mouse dorsal roots [45], whereas, in rat dorsal root ganglia (DRG), positive immunoreactivity for mTOR and S6K1 was detected mainly in the cell body of small nociceptors, coexpressing substance P or IB4 positive [49]. These data suggest that mTOR and its downstream targets are mostly transported to myelinated peripheral and central fibers in the medium and large (but not small) DRG neurons [44, 48]. In addition, at the DRG level, a predominant expression of 4E-BP1 has been detected in GFAP positive satellite cells [49]. Satellite cells are specialized glial cells that surround the cell body of sensory neurons both in DRG and in trigeminal ganglia and, like central glia, are involved in the development of chronic pain [50]. Studies from our laboratory have shown that satellite glial cells contribute to neuronal sensitization of trigeminal neurons* in vitro* [51] and that they can express functional CGRP receptors which increase the stimulatory effects of cytokines [52]. Finally, Xu et al. [49] have documented the expression of mTOR and mTORC1 downstream targets in the dorsal horn of rat spinal cord.However, these authors failed to detect the phosphorylated proteins both at the DRG and spinal cord level. On the other hand, Géranton et al. [48], using a tyramide signal amplification- (TSA-) enhanced immunofluorescent staining, demonstrated that phospho-mTOR, phospho-S6 ribosomal protein, and phospho-4E-BP1 are strongly expressed in lamina I/III projection neurons of the dorsal horn, that is, those neurons critically involved in the development and maintenance of chronic pain [53].

Consistently with a possible involvement of mTOR in chronic pain processes, it has been shown that peripheral inflammation increases mTOR activation in the spinal cord dorsal horn. In particular, intraplantar injection of capsaicin significantly increased the phosphorylation level of the S6 ribosomal protein after 2 h in the dorsal horn neurons, an effect abolished by intrathecal administration of rapamycin [48]. Intraplantar injection of carrageenan increased the magnitude of S6 phosphorylation in the ipsilateral spinal cord dorsal horn but not in the contralateral one, 4 h after treatment. In these animals, phospho-S6 together with Rheb, the positive regulator of mTOR activity, was mainly detected in neurons, but not in spinal glia (astrocytes and microglia). Moreover, basal S6 activity was also detected in larger motor neurons in the ventral horn, but it was not modified by carrageenan peripheral administration [54]. Finally, peripheral inflammation due to intraplantar injection of complete Freund's adjuvant significantly increased mTOR activation in DRG neurons, with a minor increase observed in spinal cord dorsal horn [55]. Enhanced activation of the mTOR pathway was also found in different experimental models of neuropathic pain. In adult male rat undergoing L5-L6 spinal nerve ligation (SNL), increased phospho-mTOR staining was detected in sensory neurons mainly in myelinated fibers of injured nerves [56]. However, Asante et al. [57] in the same model measured reduced levels of CGRP and S6K1 in the superficial dorsal horn neurons, just at the ligated L5-L6 nerves, but not in the uninjured L4 spinal cord. Increased mTOR activation was instead found in a mouse model of chronic crush injury (CCI) at the spinal cord level, and it was significantly reduced by intrathecal administration of rapamycin. In particular, rapamycin reduced phospho-mTOR expression at 7 and 14 days after surgery, together with significant reduction of the phosphorylation level of S6K1 and 4E-BP1 at 7 (but not 14) days after surgery [58]. In contrast, no differences in the level of mTOR activation were detected in the spinal cord dorsal horn 7 days after surgery, in a different model of neuropathic pain induced by spared nerve injury (SNI) of the sciatic nerve [48]. All together these data suggest that mTORC1 activity is significantly elevated in dorsal horn neurons in different models of chronic pain (even tough not in all models), thus suggesting a possible involvement in mediating central neuronal plasticity and thus pain hypersensitivity. In support of this hypothesis, a recent paper by Xu et al. [59] has demonstrated increased mTORC1 activation in the spinal cord dorsal horn (at the lumbar level, but not at cervical and thoracic level) in a rat model of morphine induced tolerance and hyperalgesia, thus suggesting that the reduced analgesic effects of morphine observed in long-term treatments may be due to the upregulation of mTORC1 activity within the spinal cord. It is, therefore, possible that inhibition of mTORC1 activity may have beneficial effects in chronic pain syndromes.

### 4.2. Electrophysiology

In addition to these data, there is also consistent electrophysiological evidence which point out mTOR signaling pathways as important modulators of chronic pain [44, 57, 60]. The electrophysiological analysis conducted by Jiménez-Díaz et al. [44] revealed an increased mechanical threshold of subsets of myelinated fibers, the A-fiber nociceptors, after intraplantar injection of rapamycin, following capsaicin induced injury in rats. The authors proposed that ongoing local protein synthesis is essential for the complete response of this subset of nociceptors and were pioneers in supposing the possibility that a similar process is operating at the sites of termination of sensory afferents within the dorsal horn [44]. The role of mTOR in the neuron injury-induced hyperexcitability was demonstrated later in a study conducted by Asante et al., in which rapamycin (50 *μ*L of 250 nM), directly applied on the spinal cord at L4-L5 level, was found to produce inhibitory effects on nociceptive C-fiber activity in lamina V wide dynamic range (WDR) neurons, in response to mechanically and thermally stimulus in rats after removal of lumbar vertebral segments L1–L3 of the spinal cord [60]. For the thermally induced response, a significant inhibition was only found at 35°C, with a trend towards reduction at higher temperatures. Also formalin-induced hyperexcitability was attenuated by spinally administered rapamycin, with significant effects only in the second phase of formalin test, which is thought to be due to central sensitization of dorsal horn neurons as a result of the initial attenuation of input from nociceptive C-fiber afferents occurring during the first phase. The same authors demonstrated similar effects of anisomycin, a general inhibitor of protein translation, on the formalin test, confirming that these effects were indeed due to inhibition of mRNA translation [60]. In a second paper, Asante et al. demonstrated the spinal effects of the rapamycin analogue CCI-779 on neuronal responses by using* in vivo* single-unit extracellular recordings from spinal cord neurons of rats following L5-L6 SNL [57]. The authors found that spinal administration of 250 *μ*M CCI-779 significantly attenuated specific neuronal responses to mechanical stimuli from SNL rats compared to predrug responses and sham rats, whereas no effect was established on thermally evoked responses. In particular, CCI-779 inhibited C-fiber-mediated transmission onto WDR neurons. A further significant inhibitory effect was seen on WDR neuronal postdischarge and on “wind-up” phenomenon, a potentiated response mediated by nociceptive C-fibers activity and a measure of central hypersensitivity mechanisms. The limitation of this study is represented by the fact that no differences in electrophysiological responses were found between sham and neuropathic rats before drug injection. The authors stated that the increased excitability of L4 WDR neurons in neuropathic rats could have masked the adjacent L5 ligation, according to a previous study in which periphery connected spinal neurons were shown to expand their receptive fields after the same nerve ligation [61]. The observation that CCI-779 was quite ineffective on neuronal responses in the absence of nerve injury [57] allows to confirm that mTOR signaling at the spinal level is an integral element of nociception and that its role in central sensitization likely contributes to the persistence of pain.

### 4.3. Behavioral Studies

The antinociceptive effects of rapamycin and its analogous CCI-779 (thus mTOR inhibition) have been documented in several experimental models of inflammatory and neuropathic pain. Using the formalin test, it has been shown that rapamycin (administered both intrathecally or locally in the paw) significantly reduced the second phase of behavioral pain in mice [62]. Similarly, in adult rats, intrathecal administration of rapamycin produced a significant inhibition of formalin-induced second phase flinches [63]. The formalin test is a well-characterized behavioral model of chemically induced pain consisting of two consecutive pain behavior phases, of which the second one has been suggested to involve central sensitization of the nociceptive system [64], thus suggesting an involvement of spinal mTOR in inflammation induced hyperalgesia. On the other hand, rapamycin, injected directly through the skin at L5-L6 spinal cord level 30 minutes before the formalin test, was shown to significantly reduce both pain behavioral phases of the formalin test in adult rats, indicating an involvement of mTOR in peripheral sensitization as well [60]. A possible role of mTOR kinase (and thus local protein translation) in mediating both peripheral and central neuronal sensitization is also suggested by other studies based on different models of inflammatory pain. Inflammatory pain can be induced by intradermal injection of other nociceptive and algogenic substances including capsaicin, carrageenan, and the complete Freund's adjuvant (CFA) [64]. For example, the injection of capsaicin produces both peripheral sensitization of C- and A*δ*-nociceptors and central sensitization of dorsal horn neurons [48]. The hallmark of peripheral sensitization is represented by increased thermal sensitivity, most likely supported by ERK activation in the cell body of nociceptors followed by synthesis of TRPV1 receptors and their transport to the axon terminals in the inflamed cutaneous tissue, whereas increased mechanical sensitivity is mainly due to central sensitization of spinal cord neurons [65]. In this experimental model (in adult rats), rapamycin administered either centrally or locally significantly reduced mechanical hyperalgesia without affecting thermal hypersensitivity in adult rats [44, 48]. In a similar manner, local and central injection of CCI-779 reduced mechanical hyperalgesia, but not thermal hypersensitivity developing in response to intradermal injection of carrageenan in adult mice [45]. The mechanical hypersensitivity that occurs in the undamaged area surrounding the site of injury, in these models, is known to be mostly transmitted by A-fibers and amplified by sensitized dorsal horn neurons.

In this regard, electromyographic studies have shown that both local and intrathecal injection of rapamycin significantly increased threshold temperatures for paw withdrawal evoked by fast heat ramps (activating A-fiber nociceptors) compared to control injections of vehicle, thus suggesting a direct effect on this subset of myelinated nociceptors known to be important for the increased mechanical sensitivity that follows injury [44, 48]. However, intrathecal injections of rapamycin inhibited the activation of downstream targets of mTOR in dorsal horn and dorsal roots, thus suggesting a modulatory effect on both primary afferents and central neurons [48]. Consistently with these observations, centrally administered rapamycin was shown to reduce mechanical allodynia in several models of inflammatory pain [54, 55, 59]. However, these studies reported variable effects on thermal sensitivity, with inhibitory effects observed after intraplantar injections of carrageenan [54, 63], and minor albeit significant reductions of thermal hyperalgesia induced by intraplantar injection of CFA [55]. Considering that the expression of mTOR and other components of the translational machinery has not been detected in C-nociceptors, these results further suggest a direct role of spinal mTOR in the modulation of central pain processing. Interestingly, the PI3K inhibitor, wortmannin, was more effective at reducing pain hypersensitivity in response to carrageenan, thus suggesting the involvement of other pathways (including ERK) in the development of central sensitization [63]. Moreover, intradermal injection of carrageenan increased the phosphorylation level of AKT at Ser_473_ in the dorsal horn of spinal cord thus suggesting a concomitant activation of mTORC2 in parallel with development of hyperalgesia in response to peripheral inflammation [63]. Therefore, wortmannin by inhibiting PI3K can simultaneously affect both mTOR complexes and other signaling pathways (including ERK; as described in Sections 2 and 3) potentially important in mediating peripheral and central sensitization thus resulting more effective than rapamycin in chronic pain.

mTOR inhibitors also display beneficial effects in several models of neuropathic pain, which are also characterized by the development of secondary hyperalgesia. In summary, intraplantar or intrathecal administration of rapamycin significantly reduced mechanical allodynia developing after SNI in rats [44, 48]. This model consists in a tightly ligation followed by distal sectioning of the common peroneal and tibial branches of the sciatic nerve, with preservation of the sural nerve. It is characterized by increased mechanical sensitivity observed 6 days after surgery in the spared sural territory, that is, the lateral part of the hind paw [44, 48]. Similarly in mice, intraplantar or intraperitoneal injection of CCI-779 significantly reduced mechanical allodynia observed in the lateral part of the hind paw three days after surgery [45]. Interestingly, in this study, the authors also evaluated the effect of repeated administration of CCI-779 (4 injections every 24 hours) observing a persistent reduction in mechanical allodynia which was gradually lost 48 hours after the last administration [45]. Moreover, in agreement with a possible role of mTORC2 in mediating central neuronal plasticity, the dual mTOR inhibitor Torin 1 appeared to be more effective than rapamycin in this experimental model, either after one injection or after repeated administrations [45]. Interestingly, daily administration of metformin (which also inhibits mTOR by promoting AMPK activation (see Section 2)) reduced mechanical allodynia in a mouse model of SNI [56]. In addition to these data, mTOR inhibitors were found to exert beneficial effects in other models of neuropathic pain. For example, CCI-779 or metformin significantly reduced mechanical allodynia in a model of persistent pain caused by SNL [56, 57], and rapamycin reduced mechanical hypersensitivity in mouse models of CCI [58, 66]. In agreement with these observations, Cui et al. have shown, using adult female Sprague Dawley, which chronic (14 days) intrathecal administration of rapamycin reduced both the mechanical allodynia and the thermal hypersensitivity induced by CCI [67]. However, variable effects of rapamycin on thermal sensitivity have been reported in this experimental model as well [58], suggesting a main role of mTOR in the regulation of central sensitization. Notably, peripheral nerve injury can induce a different spectrum of glial (both microglia and astrocytes) activation in the dorsal horn of the spinal cord. These cells can release inflammatory and pronociceptive mediators, thus significantly contributing to neuronal sensitization and to the establishment of chronic pain syndromes [5]. Recent experimental evidence from our group and others suggest a direct role of mTOR in the regulation of glial inflammatory responses and a potential beneficial role of rapamycin in neuroinflammatory based diseases [8], including chronic pain syndromes. In fact, rapamycin (centrally administered) reduced CCI induced astrogliosis [67] but did not modify microglial activation when injected peripherally in the affected hind paw [66]. These data suggest that rapamycin may have multiple cellular and molecular targets that can contribute to the therapeutic effects observed* in vivo*. In this regard, Marinelli et al. have demonstrated the critical role of autophagy, particularly in Schwann cells (SCs), in reducing neuronal damage, clearing myelin debris, and facilitating neuronal regeneration after injury [66]. In these animals, rapamycin significantly increased the autophagic flux in SCs, their proliferation, and improved myelination in injured nerves [66]. Consistently, rapamycin improved myelination in explant cultures from neuropathic mice by activating autophagic mechanisms [68]. However, mice lacking mTOR in SCs display hypomyelinated sciatic nerves [69], further underlying the relevance of the mTOR pathway in the regulation of myelination in both peripheral and central nervous system [70, 71].

In this field, recent evidence from our group suggests that mTOR inhibitors can reduce signs of neuropathic pain in a chronic model of a demyelinating disease, the experimental autoimmune encephalomyelitis (EAE). We have shown that chronic administration of rapamycin was able to increase the sensitivity threshold for mechanical allodynia, which is usually reduced at the clinical onset of disease [72]. In this study, we observed that rapamycin ameliorates clinical and histological signs of EAE when administered to already ill animals, at the peak of disease (therapeutic approach). Interestingly, the histological study of the brains at the end of the experiment revealed a significant improvement in the myelination of the corpus callosum in the rapamycin treated animals, which may be the consequence of reduced neuroinflammation as well as a direct effect of rapamycin [72]. In addition, rapamycin was also found beneficial in a mouse model of cancer metastatic pain. In fact, repeated treatment with rapamycin reduced both thermal and mechanical hypersensitivity developing in response to intratibial injection of prostate cancer cells. In this model of chronic cancer pain, rapamycin was also effective when administered in a therapeutic manner, that is, once daily 5 days after injection of cancer cells within the tibia [73]. Rapamycin was also effective in a similar model of metastatic cancer pain induced by intratibial inoculation of breast cancer cells [74]. Finally, rapamycin reduced hyperalgesia associated with chronic administration of morphine in rats [59], thus further supporting a potential clinical use of mTOR inhibitors in the management of chronic pain syndromes.

### 4.4. Preclinical and Clinical Evidence of a Pronociceptive Role for mTOR Inhibitors

Despite the promising results from the preclinical studies reviewed above, the role of rapamycin and rapalogs in the clinical treatment of chronic pain is undermined by the clinical evidence that chronic treatment of patients with these mTORC1 inhibitors is associated with increases in the incidence of pain [75, 76], including the possible development of complex regional pain syndrome (CRPS) [77]. In addition, the anticancer agents, RAD001 or AP23573, are associated with a number of unique toxicities, with one of the most significant being the so-called painful mTOR inhibitor-associated stomatitis (mIAS) [78]. However, mechanistic data are lacking concerning whether and how rapalogs are linked to the development of pain in patients chronically treated with these drugs; hence more and appropriate clinical studies are necessary to clarify this important issue. In this regard, the only preclinical study carried out in C57bl/6 mice that show possible negative effects of long-term mTOR inhibition with respect to pain hypersensitivity is a recent paper from Melemedjian et al. [79]. These authors demonstrated that chronic rapamycin treatment (intraperitoneally injected for 9 days) induced mechanical allodynia in sham-operated animals, while reducing mechanical hypersensitivity in SNL animals in agreement with data reviewed in Section 4.3. Similarly, rapamycin partially reversed mechanical allodynia in mice with SNI, while producing mechanical allodynia in sham animals. Chronic administration of rapamycin appeared to increase ERK and AKT phosphorylation in sham animal sciatic nerves, thus suggesting that the abrogation of the negative feedback loops on these other pathways due to the incomplete blocking of mTORC1 (see Sections 2 and 3) may cause activation of other signaling pathways responsible for pain development [79]. For example, increased ERK activity can induce sensory neuron sensitization, mechanical hypersensitivity, and spontaneous pain. Interestingly, the clinically available antidiabetic drug metformin, which is also a AMPK activator thus mTOR inhibitor (see above), prevents rapamycin-induced ERK activation and the development of mechanical hypersensitivity and spontaneous pain [79]. These data suggest that a more complete inhibition of the mTOR pathway can overcome these side effects of rapamycin and its analogs. In this regard, in a retrospective study conducted on diabetic patients, the use of metformin has been found significantly associated with reduction of pain symptoms in patients affected by lumbar radiculopathy, a very frequent form of chronic pain syndrome [80, 81].

## 5. Conclusions

Data presented in this review paper strongly suggest that mTOR has a critical role in several mechanism of pain processing, including a role in the development of chronic pain. However, clinical evidence suggests that chronic use of first generation mTOR inhibitors may be associated with development of pain hypersensitivity, thus underlying the involvement of other signaling pathways including PI3K downstream effectors (ERK, AKT, and more interestingly mTORC2). A more comprehensive understanding of these signaling pathways may lead to improved treatments for the management of chronic pain. A vast array of novel inhibitors, with a broader range of activity, is becoming available for clinical testing. These have been mostly developed for cancer treatment, but may also be employed in the management of chronic pain.

## Figures and Tables

**Table 1:** mTOR inhibitor drugs.

Classes	Drugs	mTOR (*in vitro* kinase IC50)	mTORC1 (cellular potency EC50)	mTORC2 (cellular potency EC50)	Class I PI3K (*in vitro* kinase IC50)	References
	Rapamycin	1.74 *μ*M (2 nM^1^, in presence of FKBP12)	0.4–3.5 nM^2^			[82–84]

Rapalogs	RAD001		0.4–3.5 nM^2^			[82]
	CCI-779	1.76 *μ*M	<20 nM	10–20 *μ*M		[84]
	AP23573		0.2 nM			[85]

ATP-competitive mTOR inhibitors (*first generation*)	KU-0063794	2.5 nM^1^	660 nM^3^	240 nM^3^	>5.3–>30 *μ*M	[86–88]
	PP242^4^	8 nM		300–400 nM	0.10–2.2 *μ*M	[89, 90]
	PP30^4^	80 nM			0.68–5.8 *μ*M	[89]
	Torin 1^4^	4.3 nM	2–10 nM	2–10 nM	0.17–>10 *μ*M	[90, 91]
	WEY-600^4^	9 nM^1^	300 nM	1 *μ*M	1.96–8.45 *μ*M	[92]
	WYE-354^4^	5 nM^1^	300 nM	1 *μ*M	1.89–7.37 *μ*M	[92]
	CC214-1	2 nM	40 nM	18 nM	1.38 *μ*M	[93]
	OSI-027^4^	4 nM			0.42–>30 *μ*M	[94]
	X-387^4^	23 nM^1^			0.12–>0.3 *μ*M	[95]

ATP-competitive mTOR inhibitors (*second generation*)	AZ8055	0.13 nM^1^	27 nM^3^	24 nM^3^	3.2–18.9 *μ*M	[42, 88]
	AZ2014	2.8 nM^1^	200 nM^3^	80 nM^3^	3.8–>30 *μ*M	[88]
	INK128/MLN0128^4^	1 nM	<10 nM	<10 nM	0.22–5.29 *μ*M	[96]
	WYE-125132	0.19 nM^1^	20 nM	200 nM	1.18–>10 *μ*M	[97]
	CC214-2	106 nM	386 nM	315 nM	>30 *μ*M	[93]

ATP-competitive mTOR/PI3K dual inhibitors	Wortmannin	0.2 *μ*M^1^			0.1 nM	[83, 98]
	LY294002/SF1101^5^	1.5 *μ*M^1^			0.5–1.6 *μ*M	[83, 99–101]
	PI-103^5^		*In vitro* kinase IC50: 20 nM	*In vitro* kinase IC50: 83 nM	2–15 nM	[40, 100, 101]
	Torin 2	2.81 nM	0.25 nM	10 nM	4.68–17.5 nM	[102, 103]
	GSK2126458	ND	Low nM	0.18–0.41 nM	0.04 nM	[104]
	NVP-BEZ235^5^	20.7 nM	<250 nM	8 nM	4–75 nM	[43, 101]
	NVP-BGT226^6^				4–63 nM	[105]
	SF1126 (RDGS conjugated SF1101)				Not significant inhibitory activity until hydrolyzed to SF1101	[106]
	PKI587	1.4 nM^1^	<30 nM	<10 nM	0.6–8 nM	[107]

*In vitro* mTOR kinase IC50 was evaluated using either the immunoprecipitated or the recombinant full length enzyme. Cellular potency for the two different mTOR complexes was calculated after short term incubation, ranging between 30 min and 2 h, of different cell lines with mTOR inhibitors and subsequent analysis of the phosphorylation status of specific mTORC1 (S6K or S6) or mTORC2 (AKT, at Ser_473_) substrates. *In vitro* PI3K and PIKK IC50 were measured using specific biochemical assays.

^1^A truncated mTOR enzyme was used in the *in vitro* kinase assay.

^2^Cellular potency was evaluated by inhibition of cell proliferation, using vascular smooth muscle cells stimulated by fetal calf serum.

^3^Cellular potency was evaluated using a high throughput immunocytochemical assay, carried out in the MDA-MB-468 cell line.

^4^Ratio between PI3K and mTOR IC50 is <500.

^5^The reader should also consider IC50 values reported by Hayakawa et al., 2007 [99], and Kong et al., 2009 [101].

^6^NVP-226 is considered a dual mTOR/PI3K inhibitor. However, *in vitro* preclinical data on the mTOR inhibitory activity for this compound were not found through Medline Search.
